# GM trust shaped by trust determinants with the impact of risk/benefit framework: the contingent role of food technology neophobia

**DOI:** 10.1080/21645698.2020.1848230

**Published:** 2020-12-28

**Authors:** Sumran Ali, Muhammad Asim Nawaz, Muhammad Ghufran, Sumaira Nazar Hussain, Aljaifi Saddam Hussein Mohammed

**Affiliations:** a School of Public Affairs, University of Science and Technology of China, Hefei, China; b Department of Economics and Law, Sapienza University of Rome, Rome, Italy; c Lyallpur Business School, Government College University, Faisalabad, Pakistan; d Department of Economics and Law, Wuhan University, Hubei, China; e Sana’s University, Rome, Yemen

**Keywords:** Perceived risk and benefit, trust in institutions, trust in technology, revealed information, GM knowledge

## Abstract

The present study is comparative in natures that focus on understanding the factors that influence the GM food trust level in the BRA framework and food technology neophobia in China and the USA. For this purpose, we collected 300 and 350 valid responses, respectively, through a structured questionnaire. By carefully evaluating the above relationships, we found that trust determinants such as institutional trust, technology trust, information revealed with GM food vary across both datasets. However, GM knowledge has a better association with GM food trust in both cases. Apart from this, the food technology neophobia slightly moderates the benefits-risk perception of consumers and GM trust. This study guides the policymakers to enhance GM knowledge, as GM food is scientifically proven safe for health and environment and can be a financial incentive for the farmers. Further, the study also provides direction for corporate managers to design effective marketing and communication strategies in two different countries by investigating GM food trust’s primary motivators in both nations.

## Introduction

Trust has been widely acknowledged for its central role in establishing and maintaining close, cooperative, and productive relationships.^1,2^ We focus on the conative aspect of trustworthiness, i.e. a behavioral intention of consumers to trust on genetically modified food. Similarly, consumer intentions are one of the most favorable predictors of actual buying of genetically modified food – that is consumer confidence and trust in emerging technologies such as genetic modification which is the consumer recognition of their acceptance.^3^ It is tremendously difficult for scholars and legislators to keep reliance on two equally important goals. “Genetically modified food and consumer trust” often go in the opposite direction, privileging mission over financial viability. Suppose consumer trust declines in a particular arena of genetically modified food. In that case, there may be costs to be paid in terms of the regulatory institutions involved political exposure, the industrial sector’s economic vulnerability to invest in the technology of GM food, and the potential escalation of critical media concern. The consumer trust on GM food is a useful indicator of the possible success of emerging technology^4^ not just in the region of movement directed, but also in the institutions advancing and controlling the innovation, and in the data given by these institutions to the advantage of public.^5,6^ The empirical investigation of consumer trust has provided diverse results in the context of GM food. The acceptance of new technologies has often been anticipated to be primarily based on consumer cognition of the associated risks^7^ and that risk perception is influenced by confidence in various information sources.^8^ This research examines the GM trust in the context of the USA and China against its antecedents in different cultures and possible consumer reaction. Moreover, derive practical and theoretical strategies for building GM trust in society.

Previous research has provided a partial explanation of how to incorporating the benefit-risk framework and antecedents of trust in GM food. Some scholars argue that plant and animal GM foods pose unknown health risks and a severe environmental threat.^9^ Another concern is that transgenic crop patents and intellectual property rights may result in market dominance and pricing of monopolies.^10^ Eventually, GM food success will depend on consumer trust in GM food and government approval and market adoption. Therefore, trust in public and private organizations seems to be the primary factor in determining consumer perception and attitude toward GM food. We contend that it is imperative to develop a perspective that helps to achieve GM trust. GM foods are widely recognized as a solution to present hunger problems in the third world and upcoming food shortages which would happen alongside climatic change.^11,12^ To an extent, GM trust in public, private organizations and governmental institutions has theorized as consumer trust – that conceptualized as a mixture of consumer interest, honesty and organization capabilities and transparency^13^ in relating to the GM food. Consumer attitudes toward genetically modified organisms and GM food are normally low and vary depending on the kind of organism, media coverage and propaganda food technologies.^14^

We address the research question to hypothesize antecedents of trust: trust in institutions, trust in technology, revealed information and perceived knowledge mediating by perceived risk and benefit and the role of food technology neophobia as a perceived gap between genetically modified food and consumer trust. The antecedents of trust with the integration of perceived risk and benefit have a substantial impact on achieving GM trust in society in the context of different countries. Prior research suggests that GM food’s different acceptability levels across countries are associated with knowledge of GM technology and trust.^15^ For instance, Europeans are generally less supportive of GM food but now trend gradually changing, consumer’s concerns had been decreased from 63% in 2005 to 27% in 2019; still, they far lack from the USA, Argentina, Canada, Brazil and China.^16^ Regulatory authorities in Europe for GM food have not fulfilled the criteria of legal certainty, nondiscrimination, and scientific adaptability compared to the USA and other top-five cultivating GM crops.^16^ However, the story in the USA and China is varying in the context of GM trust; there is limited research on GM trust incorporating the risk-benefit framework in the context of two countries.

Our study makes three major contributions. First, it goes beyond the extant research that primarily develops the theoretical approach that incorporating BRA (Benefits-Risk framework) and antecedents of trust in GM food. Second, we employ the idea of trust, which is strong positive feelings related to consumer involvement in business activities such as genetically modifies food that is meaningful and significant to the individual self-identity. Third, the moderating role of food technology neophobia varies from the perspective of different regions and countries in the context of genetically modified food trust that enables us to make the connection of GM food trust to community intentions.

## Theoretical Model and Hypothesis

**Figure 1.**
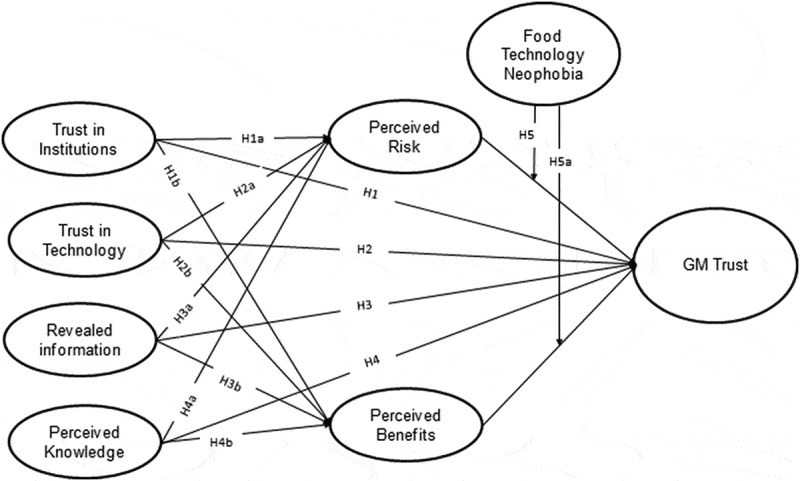
Theoretical framework

We propose a theoretical model and hypothesize a relationship between incorporating BRA (Benefits-Risk framework) and antecedents of trust in GM food, food technology neophobia, and GM trust, using comparative analysis as perceived by the USA and China see Fig. 1. The concept of trust is differently operationalized by different scholars^,17,18^ because of its various and interesting aspects, specifically in the field of GM food.

### Trust in Institution: GM Trust

There are many public and private organizations are producing GM food all over the world to competing hunger problems. In terms of rules and regulations, GM foods are similar to the natural food produced by traditional means if GM does not alter the nutrition values of the food.^19^ In the world, various regulatory institutions certify the food quality and nutrition values in the food to ensure the people health and ecological system from toxic chemicals. People trust in institutions because of the institution’s ability, benevolence, and integrity. The ability of institutions means expertise and competence in terms of varieties of goods and services. Benevolence is the goodwill of institutions for fulfilling the needs of the customers without any harm. Here, intentions and motives of the institutions play a central role: On one hand, the customer believes that institutions are entirely interested in business goals and wealth maximization without considering the possible consequences for the customer that arise the uncertainty among the consumers about health and ecological system,^20^ therefore, consumer trust in institutions is not developing in the case GM food and crops. On the other hand, when institutions^1^^1.^Institutions can be (farmers, food industry, State and media, consumer and environmental organizations, universities and scientists, GM manufactures) according to 21.Gaskell G, Allum N, Bauer MW, Jackson J, Howard S, Lindsey N. Climate change for biotechnology? UK public opinion 1991–2002. 2003. follow the high personal traits to consider all possible reservation of customer care to support the environment with friendly products and services and these traits build a favorable relationship between customer and institution which may lead to emerging the trust in instructions.^21^ The existing body of literature exhibits that consumer trust in GM context is a set of complex characteristics.^22,23^ Thus, the study investigates that institutional trust may help to build consumer trust in GM food. So we suggest:

***Hypothesis 1:****Trust in institutions has a positive impact on consumer intentions to achieve GM trust.*

### Trust in Technology: GM Trust

Prevailing literature shows that higher the trust in technology higher the intentions to adopt.^24^ In medical science, people react positively to technological advancements,^25,26^ similar passion is observed in the pharmaceutical industry.^27^ It has been appreciated by GM technology in the area of pharmaceutical research^28^ in the same way, GM technology in GM food also getting close attention across the world.^4^

Moreover, trust in technology is the organizational structural ability to control and monitor the safe use of technology in the food business.^29^ Similarly, the USA and China have several institutions that apply the restrictive rules and regulations on the originality of goods to ensure the safe use of the technologies in the food and crop industry to protect the ecological system and consumer health. On the other hand, consumer lacks the appropriate knowledge, skills, and expertise required to evaluate the GM technology, in a food context.^30^ Therefore, consumers are not sure regarding the technology intervention and its negative impact on originality, nutrition, and utility of GM food items.^31^ The existing literature exhibits that consumer trust in GM food is a set of complex characteristics^22,23^ in the absence of appropriate knowledge about GM food benefits such as to compete for the hunger problem in the entire world in upcoming decays with increasing population. Similarly, trust in technology might influence the consumer's overall trust in GM food. Based on these arguments, we suggest a hypothesis:

***Hypothesis 2:****Trust in technology has a negative impact on consumer intentions to achieve GM trust*

### Revealed Information: GM Trust

Revealed information on genetically modified food products is essential to emerging the trust of consumers to accept GM food. Therefore, it was argued that the customer would put more energy to seek alternatives to minimize food safety concerns.^32^ Revealed information is also one of the most important determinants of building the trust of GM food about the quality and safety of the food which they are consuming. Whereas, revealed information helps the consumers to control the many health issues with the help of nutrition values which are mentioned on the products, for instance, obesity challenges because of the higher caloric intake^33^ without knowing the labeling. GM food with revealed information is considered more appealing and rich in content,^34^ policymakers focus on environmental and food policy approaches, including mandated calorie menu labels for GM food products, that influence consumer choice. Moreover, GM food revealed information perceived as healthier and quilted food as compared to the food product without labeling.^35^ Revealed information influence the consumer’s perception to make the decision regarding the adoption of GM and assume it is hygienic food for health. The prior research assists us to investigate the relationship among the revealed information and GM trust perceived by consumers. Therefore, we propose that:

***Hypothesis 3:****Revealed information has a negative impact on consumer intentions to achieve the GM trust*

### Perceived Knowledge: GM Trust

Perceived knowledge of GM food means consumer knowledge about quality and nutrition values of GM food, which makes you select the more hygienic food as compared to traditional food. Consumer trust in GM food depends on the prior knowledge of consumer which provides evidence that more knowledgeable consumer about the organic food products has a better understanding of GM food which leads to emerging the trust on GM food.^36,37^ Apart from this many researchers also explained that knowledge has a strong positive impact on consumer attitude and purchase intentions of organic food in Taiwan, Malaysia,^38,39^ similarly perceived knowledge is considered as a critical element in building the GM food trust between the customers.^39^ The knowledge about every aspect (is healthier, tastes better, environmental concern, concern over animal welfare, supports the local economy and helps to sustain traditional cooking, concern over food safety, is wholesome, reminiscent of the past, and fashionable, rejection of high prices) of GM food reduces uncertainty, doubt among the consumers^36^ to enhance the understanding of GM food and also assist them in selecting the appropriate food product for their body type. Consumers with better GM food knowledge offer trust in GM food adopt in comparison to those with less knowledge. This improvement in GM food trust leads the consumer to recognize food products while making purchase decisions. Therefore, the present article makes an effort to explore the perceived knowledge of GM food is an effective predictor of GM trust. So, we suggest that:

***Hypothesis 4:****Perceived Knowledge has a negative impact on consumer intentions to achieve GM trust*

## Mediating Effect of Perceived Risk and Benefits

### Perceived Risk: GM Trust

Prior research has shown that the perceived risk depends on three factors: (1) unexplained anxiety, (2) product trust, and (3) the number of people at risk. It also has been addressed in GM food in many studies.^40^ In the same way, researchers also have divided and some of them explained the perceived risks of food safety, health and environmental concerns caused by GM food. Such as Pattanapomgthorn, Sutduean and Keohavong^41^ and Pino, Amatulli, De Angelis and Peluso^42^ found that GM foods are extended significantly by the dominant scientific methods, which have modified farming techniques that directly or indirectly affect environmental impacts. Pattanapomgthorn, Sutduean and Keohavong^41^ also explain that food protection is related to hazards such as impurities, chemical substances, toxins and diet drawbacks and also linked with culture, religion and family. Therefore, many institutions in the world are working to ensure food safety, health and overcome the environmental concerns.^20^ Each country has a chain of protocols to determine the authenticity, reliability and safety.

Whereas, institutional trust is a vital component to ensure food safety and reduced the fake rumors about GM food through increasing GM knowledge. While, the current focus is on the perceived risk of GM food, which permits the author to discuss institutional trust not only as an abstract concept but also provides freedom to make individual intentions into the acceptance of GM food. In the GM context of consumption and trust, we develop a strong understanding of the acute social dynamics and interests that drive the controversies and difficulties of research in the GM food sector.^13^ Even individuals assume that genetically modified food is associated with relatively high-perceived risk and unknown consequences, but they do not reject genetically modified food. The adoption of GM food varies according to the kind of application. Generally, these applications are more preferably considerable in plants as compared to the animals. Moreover, individuals consider GM food more negatively then genetically transformed drugs. We propose perceived risk is a potential gap between trust in institutions and GM trust. The potential for risk in using GM foods remains just that – potential. There has yet to be an event that would allow institutions and experts to move GM food from an uncertain risk to a quantifiable hazard. Therefore we suggest that

***Hypothesis 1a****: Trust in institution is negatively related to the consumer intentions to achieve GM trust through perceived risk.*

Prior research has shown that the public’s attitude to technology or a food product is essential for technological development in food products and commercialization of it.^43,44^ It also has generally assumed that people consider riskier technological innovations in food products and less likely to accept them.^45^ However, they cannot process and evaluate the scientific risk involved in technological innovation in food products even they cannot assess and process this complicated mechanism. Next, the individual has a specific socio-economic, cultural and psychological characteristic that might influence the individual perception to adopt the GM food at same risk level. The future acceptance of GM technologies is heavily dependent on consumer perception. GM technologies acceptance varies according to its application.^23^ The GM-based development in the medical and textile sector is rather welcomed in comparison to its enactment in the food sector.^22^ The researchers agree mostly that GM technology acceptance perception is based on the consumer perceived risks.^22,23,46^ Higher the risks lower consumer acceptance, lower the risk higher the consumer acceptance. Similarly, consumer acceptance is dependent on the level of trust.^23^ As consumer trust increases, risks deteriorate to the minimum level.^46^ GM food is a controversial segment surrounded by rumors and fake news^47^; it is wrathful to study trust in technology and its ability to influence the perception of the risk.

***Hypothesis 2a****: Trust in technologies is negatively related to consumer intentions to achieve GM trust through perceived risk.*

Previous studies have shown various factors that influence consumer perception and action, which become the source of trust. Revealed information is one factor that has gained importance in playing a critical role in forming consumer trust^48^ that is also perceived risk because of the negligible risk may alter public perception into intense feeling toward GM food. For example, if GM food labeling does not have the same effect which is mentioned on the product, consumer trust would lose in genetically modified foods and scatter misleading reports on GM foods that could harm the goodwill of GM foods. Several studies can be cited that confirmed the certification and revealed information role in promoting the interest of consumers in the adoption of GM food.^49^ Miller and Cassady^50^ concluded that consumer understanding of food’s nutritional value for consumption is connected to the frequent use of GM food labels, which might include ingredient description, as well as health and nutrition claims. Consumers follow GM food according to their own needs; for instance, some want to reduce the weight they use zero fat milk and some want fats they used fat rich milk. After the experience, they found any negative change or no change that GM food becomes a potential risk in the sense of revealed information. Therefore, it is essential to understand the individual perceptual perceived risk related to GM food. So, we purposed that

***Hypothesis 3a****: Revealed information is negatively related to the consumer intentions to achieve GM trust through perceived risk.*

Perceived knowledge from an unauthentic source such as social and digital media, internets may cause a potential risk that is associated with the internal attribution of responsibility, the social standards and the sense of guilt of the consumers. Knowledge also directly influences the consumer intention attitude toward the adoption of GM food. Perceived knowledge of consumers theoretically consists of two dimensions: familiarity and product knowledge. Familiarity means to accumulated consumer experiences, that experience could be positive, which becomes a strong belief in context GM food if negative consequences are resulting in rejection of GM food. At the same time, product knowledge refers to the sum of product class information and rules stored in an individual’s memory.^51^ Based on the theoretical foundation, the current study focuses on the perceived risk of consumers of GM food negatively influences the relationship between perceived knowledge and trust behavior to adopt the GM food – specifically, consumers’ familiarity with a product and product-specific knowledge. So we suggest that:

***Hypothesis 4a****: Perceived knowledge is negatively related to consumer intentions to achieve GM trust through perceived risk.*

### Perceived Benefits: GM Trust

Perceived benefits are ideas about favorable outcomes linked with consumer behavior to respond to a real or perceived threat.^52^ The perceived benefit is normally applied to the general buying or accepting products and is specific to an individual’s attitude to engage in a particular shopping action (GM food) that will yield stratification. Recently, there is no classification of perceived benefits of trust behavior to the adoption of GM food. There are some scholars who provide the perceived benefits regarding consumer attitude toward GM food applications for medical and health benefits, nutritional enhancement, obesity and cholesterol control food.^53,54^ Moreover, some scholars provide the perceived benefits regarding consumer behavior, for instance^55^ includes seven key perceived benefits three for online buying behavior (price, convenience and recreational benefits) and four for online shopping (shopping convenience, the comfort of shopping, product selection and enjoyment). Kauffman, Lai and Ho^56^ explore online group auctions sequence-based, time-based and quantitative incentives, and consumer fairness perceptions.

Trust in an institution or someone else has a critical effect on perceived benefits. According to Siegrist,^57, 58^ institutional trust in GM technology reduces the effect of perceived risk and also enhances the perceived benefit of GM technology. On the other hand, institutional credibility, integrity and benevolence play vital roles to reshape consumer perception to accept GM food because sometimes individuals make a judgment based on the institution’s credibility to select the GM food without having appropriate knowledge. People trust in institutions, organizations, gene technology because of personality traits, self-interest and rational prediction.^59–61^ Similarly, trust in organizations and experts performing gene transformation and manipulations had a substantial effect on the benefits perceived is taken as given in this research. Previous researches are providing sufficient knowledge to link the perceived benefits with antecedents of trust in GM food and GM trust. Thus we theorize that

***Hypothesis 1b****: Trust in institutions is positively related to consumer intentions to achieve GM trust through perceived benefits.*

Studies examining the public perception of innovative technologies show that public trust in technological advancement is one prime acceptance factor.^23,24^ In general, the public seems to be less optimistic about GM food technologies compared to other sectors.^30^ In this era of internet and social networking sites, an ample amount of negative information is fallowing to the consumers.^62^ Literature shows that the initial impression of technology is vital to gain consumer trust.^63^ After two decades of negative framing of food technologies, now governments, scientists and social activists have focused on potential advantages and benefits of GM food.^47^ A consensus exists between the scientific communities that GM food is as safe as ordinary food. Therefore, we derive the following hypothesis:

***Hypothesis 2b****: Trust in technology is positively related to consumer intentions to achieve GM trust through perceived benefits.*

Revealed information on GM products aims to inform the consumers about the nutrition values of food for health care. Generally, consumers use the food without knowing the nutrition values which cause various problems like obesity, skin and heart issues.^64^ The perceived benefits of revealed information on GM food strongly influence the consumer’s perception and commitment regarding the adoption of GM food. Cheung, Lau and Lam^40^ found that knowledge of organic food is one of the key factors influencing consumer attitudes to organic food consumption in Taiwan. The revealed information on GM food reduces the uncertainty of consumers and plays a supportive role in enhancing GM food understanding. It also helps to increase the repurchase of GM food, creates the dominant position of GM food in the traditional market. Considering the discussion above in the current context, we can predict that the revealed information regarding GM food for consumers is an incremental role in the trust behavior of the consumer to adapt to GM food. Thus, we hypothesize that:

***Hypothesis 3b****: Revealed information is positively related to the consumer intentions to achieve GM trust through perceived benefits.*

Perceived benefits are a dynamic cycle of consumer perception and reaction toward GM food. This dynamism may be motivated by the increasing knowledge of GM products as well as enhanced individuals’ knowledge regarding GM technologies^65^ by increasing the efficiency of their use, thereby decreasing the cost of using them. Some researches empirically have shown the direct association between knowledge and attitudes, revealing that there is a direct and positive relationship between an increasing knowledge of GM technology and increasing support to GM applications^66^ because of increasing consumer knowledge enhance the trust behavior of the consumer to adopt the GM food. So we propose that

***Hypothesis 4b****: Perceived knowledge is positively related to consumer intentions to achieve GM trust through perceived benefits.*

## Moderation Effect of Food Technology Neophobia

Food technology neophobia refers explicitly to fear of the new or unfamiliar technology in GM foods like neophobic people have pessimistic perceptions and fewer expectations of food taste.^67^ Apart from this, many people have specific food preferences, which they usually take in daily life either, that are appropriate for a healthy body or not. The behavior of food consumption has always been a complicated subject because numerous factors can influence consumer decision making.^68^ Personal traits of consumers are essential characteristics, which have a strong influence to shape the behavior of an individual to take action to accept unfamiliar genetically modified food products. In addition, Grebitus, Steiner and Veeman^69^ identify the role of individuals personality in shaping the consumers’ willingness to accept GM food, which is a new gene technological product.

In this section, we explore the moderation role of food neophobia technology on BRA (Benefits-Risk framework) and trust behavior adoption of GM food. Many consumers are interested in the potential benefits of new food technology because of product quality, appearance, taste, and disease-preventing ability.^70^ While, some consumer is highly concerned about new GM food products and novel gene technology like agri-biotechnology, cloning, and nanotechnology.^71^ A lack of perceived knowledge and trust behavior to adopt GM food technologies has negatively influenced consumer’s perception, attitude, and decision-related to purchasing GM food by innovative technologies. The “credence qualities” of food technology, such as safety, durability, health, environmental and nature, that can lead to perceived risk, skepticism, and insecurity, especially when consumers lack trust and understanding about novel food technologies.^72^

Previous theoretical and empirical studies have shown the strong impact of FTN on consumer acceptance of food technologies’ related decision-making processes.^73^ For instance, Matin, Goddard, Vandermoere, Blanchemanche, Bieberstein, Marette and Roosen^74^ confirmed that neophobia in food technology is an essential factor in determining the risk and benefit perceptions of Canadian consumers in nanotechnology applications and that it influences the negative behavior of consumers about using nanotechnology in both general and particular contexts, such as food packaging and food production. Based on the literature evidence cited above, trust behavior to the acceptance of GM food, food technology neophobia might moderate the relationship between mediating (perceived benefit & perceived risk) and dependent variables (GM trust). So, it is hypothesized that:

***Hypothesis 5:****Food technology neophobia has a moderation role slightly on the relationship between perceived risk and consumer intentions to achieve GM trust*

***Hypothesis 5a****: Food technology neophobia has a moderation role slightly on the relationship between perceived benefit and consumer intentions to achieve GM trust*

## Methods

### Sample and Data

Our research group has been studied GM food and crops from 2018 to now. We also have been paying attention to the development of consumer attitudes toward GM food and crops. We collected the data from July 2019 to November 2019 with a structured questionnaire. We interviewed people via internet (e-mail and face to face) with the cooperation of the Center of Innovation Management of the University of Science and Technology of China (USTC), and USTC professor is working in the USA. Based on these research experiences, we have a precise understanding of Chinese and American views on GM food. The questionnaire was presented to the American and Chinese people in English and Chinese languages, respectively. In translating the questionnaire from English to Chinese, semantic equivalence was ensured through back-translation (Brislin, 1970). Form China, we collected 300 valid responses by targeting the specific provinces (Guangdong, Hainan Island, and Guangxi) which are cultivating the GM Papaya Fruit on ~8,475 ha,(Beijing, Fujian, and Yunnan) they are growing GM Petunia Flowers, Sweet pepper PK-SP01, Tomato PK-TM8805R on unknown hectares (Shandong Province) GM Corn (Variety: BVLA430101) which is not commercially approved (Hunan, Jiangxi, Fujian, Zhejiang, and Anhui) they are cultivating GM Rice which is also not approved by Government.^20^ For the USA, we successfully received 350 valid responses from USA states (Illinois, Indiana, Iowa, California, Arkansas and Michigan) which are producing major GM crops and food such as Bt-corn, Soybean, Potato, Papaya, Canola and Sugar Beet.^2^^2.^These are official US Government institutions who are providing the reliable details about GM food and crops https://www.fda.gov/food/agricultural-biotechnology/gmo-crops-animal-food-and-beyond, https://www.ers.usda.gov/data-products/adoption-of-genetically-engineered-crops-in-the-us.aspx, https://www.ers.usda.gov/webdocs/publications/45179/43668_err162.pdf We considered these specific places because of agricultural dependencies and people's understanding of GM food and crops.

Moreover, for China, we sent 1000 e-mail to the respondents most of them didn’t reply, some e-mail return back because of server failure, inactive e-mails and at the end got 320 responses, 30 responses were incomplete 20 didn’t make sense which could be outliers like filled without attention and we deleted. So via internet, we got 270 replies and 30 responses collected via face-to-face interviews during the conference which is held by the University of Science and Technology of China and the response rate was 30%. Similarly, with the help of USTC research center, we sent 500 e-mails to respondents and got 300 valid responses in the case of the USA response rate was 70%. We also target three groups of consumers: 1. Those who were already experienced the approved genetically modified food, 2. Those who are not liking genetically modified food and only trust natural food, 3. Those who have knowledge about GM food but they are using some GM food in daily life without knowing. The reason behind this methodology was to access the keen intention of consumers, real responses and to examine our model.

Table 1 summarizes the demographics of the respondents in the final sample in group 1 the age of most of the respondents ranges between 18 and 41, 60% were female, 40% were male, group 2, 24–41, 62% were females 37% males, and group 3, 24–41, 44% females and 56% males in USA context. In Chinese context, Group 1 range is 24–35, 25% were females 75% male, Group 2, 24–47, 58.3% female 41.6% males and Group 3 18–41, 39.2% female 60.7% male. Moreover, 36.2% were found male and 63.8% female.

**Table 1.** Socio-demographic analysis

	USA				China			
Group 1	Category	Respondents 200	Frequency	Percentage (%)	Category	Respondents 40	Frequency	Percentage (%)
	Gender	Male	80	40	Gender	Male	30	75
		Female	120	60		Female	10	25
	Age	18–23	35	17.5	Age	18–23	4	10
		24–29	60	30		24–29	15	37.5
		30–35	37	18.5		30–35	10	25
		36–41	28	14		36–41	8	20
		42–47	17	8.5		42–47	3	7.5
		48–53	12	6		48–53	0	0
		53 & Above	11	5.5		53 & Above	0	0
	Education	High school	8	4	Education	High school	0	0
		College	42	21		College	3	7.5
		Graduate	50	25		Graduate	19	47.5
		Masters	70	35		Masters	14	35
		Others	30	15		Others	4	10
Group 2	Category	Respondents 100	Frequency	Percentage (%)	Category	Respondents 120	Frequency	Percentage (%)
	Gender	Male	37	37	Gender	Male	50	41.6
		Female	62	62		Female	70	58.3
	Age	18–23	6	6	Age	18–23	6	5
		24–29	29	29		24–29	33	27.5
		30–35	38	38		30–35	31	25.8
		36–41	20	20		36–41	25	20.8
		42–47	7	7		42–47	14	11.6
		48–53	0	0		48–53	11	9.16
		53 & Above	0	0		53 & Above	0	0
	Education	High school	0	0	Education	High school	3	2.5
		College	8	8		College	14	11.6
		Graduate	50	50		Graduate	40	33.3
		Masters	40	40		Masters	60	50
		Others	2	2		Others	3	2.5
Group 3	Category	Respondents 50	Frequency	Percentage (%)	Category	Respondents 140	Frequency	Percentage (%)
	Gender	Male	28	56	Gender	Male	85	60.7
		Female	22	44		Female	55	39.2
	Age	18–23	0	0	Age	18–23	12	8.57
		24–29	16	32		24–29	43	30.7
		30–35	20	40		30–35	50	35.7
		36–41	14	28		36–41	27	19.2
		42–47	0	0		42–47	8	6.66
		48–53	0	0		48–53	0	0
		53 & Above	0	0		53 & Above	0	0
	Education	High school	0	0	Education	High school	11	7.85
		College	0	0		College	28	20
		Graduate	21	42		Graduate	41	29.28
		Masters	29	58		Masters	60	42.85
		Others	0	0		Others	0	0

### Dependent Variable: GM Trust

We selected five statements: for GM trust, each describing the individual beliefs on GM food sees in the appendix. They were measured using a 5-point Likert scale 1 (strongly agree) to 5 (strongly disagree) answers. We assessed the reliability and validity of all items of two countries China, USA respectively by Cronbach’s alpha 0.916, 0.816 convergent and discriminant validity (See Table 3)

**Table 2.** Exploratory factor and reliability analysis

USA					China				
Items	Loadings	CR*	α	AVE	Items	Loadings	CR*	α	AVE
Trust in Institution		0.915	0.860	0.781			0.848	0.761	0.583
TI1	0.878				TI1	0.829			
TI2	0.863				TI2	0.777			
TI3	0.911				TI3	0.769			
T14	0.543 Removed				T14	0.671			
Trust in Technology		0.948	0.927	0.821			0.897	0.829	0.744
TT1	0.882				TT1	0.880			
TT2	0.913				TT2	0.860			
TT3	0.927				TT3	0.848			
TT4	0.902				TT4	0.581 Removed			
Revealed Information		0.919	0.883	0.740			0.842	0.753	0.574
RI1	0.871				RI1	0.840			
RI2	0.870				RI2	0.726			
RI3	0.853				RI3	0.814			
RI4	0.846				RI4	0.633			
Perceived knowledge		0.910	0.852	0.772			0.851	0.738	0.655
PK1	0.904				PK1	0.760			
PK2	0.869				PK2	0.860			
PK3	0.862				PK3	0.806			
Perceived Risk		0.945	0.922	0.810			0.846	0.756	0.582
PR1	0.912				PR1	0.892			
PR2	0.915				PR2	0.833			
PR3	0.886				PR3	0.856			
PK4	0.887				PK4	0.765			
Perceived Benefit		0.917	0.880	0.735			0.849	0.736	0.653
PB1	0.871				PB1	0.751			
PB2	0.846				PB2	0.833			
PB3	0.861				PB3	0.836			
PB4	0.850				PB4	0.819			
Genetically Modified Trust		0.899	0.916	0.624			0.903	0.870	0.609
GMT1	0.829				GMT1	0.852			
GMT2	0.773				GMT2	0.848			
GMT3	0.800				GMT3	0.780			
GMT4	0.809				GMT4	0.861			
Food Technology Neophobia		0.947	0.937	0.663			0.907	0.887	0.626
FTN1	0.788				FTN1	0.807			
FTN2	0.798				FTN2	0.780			
FTN3	0.859				FTN3	0.818			
FTN4	0.843				FTN4	0.821			
FTN5	0.829				FTN5	0.848			
FTN6	0.866				FTN6	0.843			
FTN7	0.839				FTN7	0.831			
FTN8	0.824				FTN8	0.832			

Note: CR; Composite reliability, AVE; Average variance extracted

**Table 3.** Discriminant and Convergent Validity

	CR	AVE	MSV	1	2	3	4	5	6	7	8
USA											
1. Genetically Modified Trust	0.948	0.821	0.812	0.944							
2. Perceived Benefit	0.917	0.735	0.677	0.803	0.857						
3. Perceived knowledge	0.910	0.772	0.651	0.784	0.821	0.879					
4. Perceived Risk	0.945	0.810	0.645	0.803	0.841	0.748	0.900				
5. Revealed Information	0.919	0.740	0.686	0.790	0.828	0.772	0.78	0.860			
6. Trust in Institution	0.915	0.781	0.612	0.870	0.900	0.770	0.781	0.828	0.884		
7. Trust in Technology	0.948	0.821	0.654	0.779	0.810	0.807	0.803	0.759	0.782	0.906	
8. Food Technology Neophobia	0.947	0.663	0.621	0.886	0.837	0.807	0.844	0.850	0.878	0.806	0.890
China											
1. Genetically Modified Trust	0.903	0.609	0.401	0.780							
2. Perceived Benefit	0.849	0.653	0.622	0.770	0.808						
3. Perceived knowledge	0.851	0.655	0.497	0.705	0.626	0.810					
4. Perceived Risk	0.846	0.582	0.538	0.733	0.652	0.560	0.763				
5. Revealed Information	0.842	0.574	0.416	0.633	0.645	0.482	0.636	0.758			
6. Trust in Institution	0.848	0.583	0.565	0.752	0.664	0.692	0.638	0.658	0.764		
7. Trust in Technology	0.897	0.744	0.490	0.700	0.647	0.583	0.616	0.686	0.697	0.863	
8. Food Technology Neophobia	0.907	0.626	0.590	0.768	0.743	0.704	0.666	0.627	0.712	0.643	0.721

Note: CR; Composite reliability, AVE; Average variance extracted, MSV; Mean squared variance

aThreshold values for convergent validity CR>0.7, AVE>0.5, CR>AVE, for discriminant validity MSV<AVE

### Independent Variables

We employed 34 items (see in appendix) according to our perspective to calculate the institutional trust, trust in technology, perceived knowledge, revealed information, perceived risk, perceived benefit, food technology neophobia, of a respondent. Also, we used consensus among topic experts by an amended card-sorting technique to conduct the above literature review.^75,76^ It enabled us to determine content validity and also helped us to decide what items we can exclude or include.^77^ We assessed the reliability and validity of all constructs of China, USA, respectively, by Cronbach’s alpha (see in Table 2) and convergent and discriminant validity (see in Table 3).

### Measurement

#### Convergent and Discriminant Validity

We performed a reliability analysis through Cronbach’s alpha for internal consistency to test the fitness of the research model for each country. Cronbach’s alpha was from 0.937 to 0.852, 0.887 to 0.736, for the USA and China respectively, which were higher than the recommended minimal cutoff score of 0.7.^78^ We performed CFA using the AMOS 25 was carried out using a maximum probability estimate for all 350, 300 respondents in the case of USA, China respectively to assess the underlying structure of the variables in the model. All constructs were evaluated for unidimensionality, reliability and validity.^79–81^ We followed the approach to access the convergent and discriminant validity by composite reliability (CR), average variance extracted (AVE) Mean squared variance (MSV) used in.^82,83^ As shown in Table 2, all items loaded above 0.60 on their assigned factors and significantly associated with their specified constructs for each country. These results provided evidence of unidimensionality. CR values are greater than 0.7 in case of all two countries and the average variance extracted (AVE) for the measures ranged from 0.663 to 0.821, 0.626 to 0.744, for USA and China, respectively (see Table 2) exceeding the recommended value of 0.50 and confirming convergent validity.^82,84,85^ The maximum shared variance between any pair of constructs should be lower than the AVE for each structure to ensure discriminating validity.^80,86^ The AVE value of each construct for USA and China was higher than the square correlation, which indicates that the discriminating validity is achieved see Table 3. Hence, a statistically acceptable model is identified. There is no concern of convergent and discriminant validity.

#### Valuation of Model Fit

Table 4 shows the results of Standardized Root Mean Square Residual (SRMR) as a goodness-of-fit measure for PLS-SEM. The data for present study show the satisfactory goodness of fit, moreover, the China dataset shows SRMR 0.065 and the USA dataset shows SRMR 0.071, indicating that all datasets satisfy the requirements for the goodness of-fit.^87,88^We also check some others useful indicators for fitness of model which explained the acceptability and goodness of fit for USA (chi-square value (df) = 657.942 (270); CFI =. .956; TLI = .946, RFI = .913, GFI = .863; NFI = .927; RMSEA = .069) and for china (chi-square value (df) = 456.609 (223); CFI = .958; TLI = .947, RFI = .899, GFI = .896; NFI = .918; RMSEA = .059) both results are quite reasonable and acceptable.

**Table 4.** Model fit using SRMR

Saturated and Estimated Model								
Data set	Criteria	SRMR	CFI	TLI	NFI	RFI	GFI	RMSEA
USA	≤0.08	0.065	.956	.946	.927	.913	.863	.069
China	≤0.08	0.071	.959	.949	.918	.899	.896	.059

#### Results

Table 5 reports the correlation matrix of the BRA framework with antecedents of trust in GM food, moderator food technology neophobia and dependent variables of GM trust are significantly correlated. Collinearity tests have been performed, and we have seen that the multicollinearity of independent, moderator, mediator and dependent variables was not a concern for China (VIF range between 1.675 and 2.862), USA (VIF range between 1.569 and 2.268). VIF values less than three are acceptable and depict a high correlation amongst variables.^89^ The structural model defines the causal relationships among the constructs in the model.^90^ The bootstrapping method, with a re-sampling of 5000, is used to estimate the significance of the path coefficient.^90^ The path coefficients for China and USA datasets are shown in Table 6.

**Table 5.** Correlation Matrix

Constructs	1	2	3	4	5	6	7	8
USA								
1. Food Technology Neophobia	1							
2. Genetically Modified Trust	0.643	1						
3. Perceived Benefit	0.687	0.639	1					
4. Perceived knowledge	0.563	0.584	0.421	1				
5. Perceived Risk	0.597	0.603	0.541	0.748	1			
6. Revealed Information	0.601	0.690	0.628	0.772	0.780	1		
7. Trust in Institution	0.628	0.703	0.680	0.697	0.681	0.528	1	
8. Trust in Technology	0.649	0.679	0.610	0.507	0.703	0.759	0.782	1
China								
1. Food Technology Neophobia	1							
2. Genetically Modified Trust	0.678	1						
3. Perceived Benefit	0.431	0.530	1					
4. Perceived knowledge	0.504	0.605	0.626	1				
5. Perceived Risk	0.666	0.533	0.652	0.560	1			
6. Revealed Information	0.627	0.633	0.645	0.482	0.636	1		
7. Trust in Institution	0.702	0.417	0.664	0.692	0.538	0.658	1	
8. Trust in Technology	0.643	0.509	0.647	0.583	0.416	0.686	0.697	1

**Table 6.** Measurement of structural model path coefficients by bootstrapping

		USA data		Chinese data	
	Relationship	Est.	Result	Est.	Result
	Direct relationship				
H1	Trust in Institutions → GM trust (GMT)	−0.009	Rejected	−0.019	Rejected
H2	Trust in Technology → GM trust	0.013	Rejected	−0.001	Rejected
H3	Revealed Information → GM trust	−0.033*	Accepted	−0.024	Rejected
H4	Perceived Knowledge → GM trust	−0.037*	Accepted	−0.038**	Accepted
	Moderation effect of Food Technology Neophobia (FTN)				
H5	Perceived Risk →Food Technology Neophobia → GM trust	0.057*	Accepted	−0.122*	Accepted
H5a	Perceived Benefit →Food Technology Neophobia → GMT	−0.113*	Accepted	0.065*	Accepted
	Indirect relationship				
	Mediation effect				
H1a	Trust in Institutions → Perceived Risk → GM trust	−0.029*	Accepted	−0.011	Rejected
H2a	Trust in Technology → Perceived Risk → GM trust	−0.016*	Accepted	−0.002	Rejected
H3a	Revealed Information → Perceived Risk → GM trust	−0.011*	Accepted	−0.015*	Accepted
H4a	Perceived knowledge → Perceived Risk → GM trust	−0.004	Rejected	−0.011*	Accepted
H1b	Trust in Institutions → Perceived Benefit → GM trust	0.568**	Accepted	0.529**	Accepted
H2b	Trust in Technology → Perceived Benefit → GM trust	0.134*	Accepted	−0.067	Rejected
H3b	Revealed Information → Perceived Benefit → GM trust	0.139*	Accepted	0.323**	Accepted
H4b	Perceived knowledge → Perceived Benefit → GM trust	0.224**	Accepted	0.273**	Accepted
R2	Perceived Risk	0.729		0.523	
R2	Perceived Benefit	0.861		0.577	
R2	GM trust	0.950		0.792	
Q2	Perceived Risk	0.551		0.527	
Q2	Perceived Benefit	0.592		0.588	
Q2	GM trust	0.542		0.574	

*Two-tailed significance, * = *p* <.05; ** = *p* <.001

The USA and Chinese perspectives, hypothesis 1 indicates that trust in institutions did not significantly influence consumer intentions to achieve GM trust. Hypothesis 1 was rejected (β = −0.009; β = −0.019). In the case of USA, hypothesis 1a’s proposition of perceived risk negatively fully mediating the relationship between trust in the institution and consumer intentions to achieve the GM trust on the other hand, in Chinese context no mediation. Therefore, hypothesis 1a was also accepted for USA (β = −0.029; *p* < .05) rejected for China (β = −0.011). Moreover, hypothesis 1b’s proposition of perceived benefits is positively fully mediating the relationship between trust in institutions and consumer intentions to achieve GM trust. Hypothesis 1b was also accepted (β = 0.568; 0.529, *p* < .001) in both cases.

The USA and Chinese perspectives, hypothesis 2 indicates that consumer’s trust in technology did not significantly influence consumer intentions to achieve GM trust. Hypothesis 2 was rejected (β = 0.013; β = −0.001). In the case of USA, hypothesis 2a’s proposition of perceived risk has negatively mediating the relationship between trust in technology and consumer intentions to achieve the GM trust but for Chinese no significant impact on consumer intentions to achieve the GM trust. Therefore, hypothesis 2a was also accepted for USA (β = −0.016; *p* < .05) rejected for china (β = −0.002). For the USA, hypothesis 2b’s proposition of perceived benefits has positively mediating the relationship between trust in technology and consumer intentions to achieve GM trust. Hypothesis 2b was also accepted (β = 0.134; *p* < .05) and rejected for China (β = −0.067).

From the USA perspective, hypothesis 3 indicates that revealed information has a negative impact on consumer intentions to achieve GM trust but for Chinese no significant impact. Hypothesis 3 was accepted for USA (β = −0.033; *p* < .05) and rejected for China (β = −0.024). For both countries, hypothesis 3a’s proposition of perceived risk has negatively mediating the relationship between revealed information and consumer intentions to achieve the GM trust, for the USA its partial mediation while for china is full mediation and hypothesis 3a was also accepted (β = −0.011; −0.015 *p* < .05). For the USA, hypothesis 3b’s proposition of perceived benefits has positively mediating the relationship between revealed information and consumer intentions to achieve GM trust. Hypothesis 3b was also accepted (β = 0.323; *p* < .001, 0.139 *p* < .05).

USA and Chinese perspectives, hypothesis 4 indicates that perceived knowledge has a negative impact on consumer intentions to achieve the GM trust and hypothesis 4 was accepted for both countries (β = −0.037; *p* < .05, β = −0.038; *p* < .001). Hypothesis 4a’s proposition of perceived risk has negatively mediated the relationship between perceived knowledge and consumer intentions to achieve GM trust in the Chinese context and for the USA, no significant influence. Therefore, hypothesis 4a was also accepted for Chinese (β = −0.011 *p* < .05) rejected for USA (β = −0.004). Hypothesis 4b’s proposition of perceived benefits have positively influenced the relationship between perceived knowledge and consumer intentions to achieve GM trust for both countries. Hypothesis 4b was also accepted (β = 0.224; 0.273, *p* < .001).

Hypothesis 5, for USA dataset food technology neophobia positively impacts the relationship between perceived risk and consumer intentions to achieve the GM trust (β = 0.057 *p* < .05) for China negatively influenced (β = −0.122, *p* < .001), H5 is accepted in both cases. Hypothesis 5a, for USA dataset food technology neophobia negatively impacts the relationship between perceived benefit and consumer intentions to achieve the GM trust (β = −0.113, *p* < .001) for China positively influenced (β = 0.065, *p* < .05), H5a is accepted in both cases.

In behavioral research, the standardized value of R^2^ above 0.2 is acceptable.^91^ For the USA and China, the R^2^ values for perceived risk are 0.729, 0.523, for the perceived benefit are 0.861, 0.577 and for GM trust are 0.950, 0.792. Further, blindfolding procedure was adapted to examine the relevance of exogenous variables and the model performance, that is just another re-use procedure (Chin, 1998; Mikalef et al., 2017). Blindfolding method is the combination of function fitting and cross-validation, by evaluating the predictive relevance of each construct by observing the differences in criterion estimates (Q^2^) (Joe F. Hair et al., 2012). Q^2^ > 0 indicates the relevance of the model (Jr et al., 2017). Our results for USA and China of Q^2^ show that perceived risk toward GM trust (Q^2^ = 0.551, 0.527), perceived benefit toward GM trust (Q^2^ = 0.592, 0.588) and GM trust (Q^2^ = 0.542, 0.574) which are satisfactory which is above the cutoff value of 0.10. Hence, the study has satisfactory predictive relevance.

#### Discussion

The study focused on investigating the factors influencing the genetically modified food trust with the mediating role of perceived benefits and risk perception and the moderating role of food technology neophobia. It is very vital to compare China and USA because of Chinese population almost 1.4 billion and agriculture dependency on the USA. China already becomes world's largest importer of agricultural products from the European Union (EU) and the USA in 2019 almost 133.1 USD billion US dollar. Apart from this, China and the USA also have trade tensions which are creating a strong influence on trade and the USA imposing retaliatory tariffs that causing the price inflation in China.^3^^3.^U.S. Department of Agriculture, foreign agriculture services published report on September 2020 about above all statistic. https://www.fas.usda.gov/data/china-evolving-demand-world-s-largest-agricultural-import-market#:~:text=China%20is%20now%20the%20world’s,with%20imports%20totaling%20%24133.1%20billion. On the other hand, according to Statista USA has become the world's largest producer of GM food and crops in the world that covers almost 75 million hectares of USA landscape, on other side, China is just covering 2.9 million hectares with GM food and crops.^4^^4.^Statista has been published report August 2020 about Area of genetically modified (GM) crops worldwide. https://www.statista.com/statistics/271897/leading-countries-by-acreage-of-genetically-modified-crops/#:~:text = Global%20genetically%20modified%20crops%20by%20countries%202018%2 C%20based%20on%20acreage&text = The%20United%20States%20had%20the,little%20over%2051.3%20million%20 hectares. China has also become the largest importer of GM food (GM soybeans and canola) from the USA that is the world's biggest producer.^5^^5.^https://www.cnbc.com/2019/12/31/china-approves-two-new-genetically-modified-crops-from-us-for-import.html That’s why Chinese Government is spending more money on research and development of GM food to promote into the general population to increase GM trust and reduced the agriculture imports.

This comparative study offers interesting findings that explain the public understanding, ethnocentrism and animosity attitude of consumer willingness to consume GM food, and an opportunity to policymakers to develop strategic choices to give possible alternatives for consumers to choose the best option from the food. Whereas, China, we found the perceived knowledge is the only predictor of GM food trust and their interaction terms were also significant. These findings support the previous results of^92–94^ which establish that GM food perceived knowledge among consumers is having the better predictive ability of consumption trust. On the other hand, revealed information and perceived knowledge were found significant predictors of GM food trust in the US context. The findings regarding information revealed are different from,^95,96^ which found a non-significant relation. Information disclosure is a hot topic in the western world and consumer rights organizations are constantly pushing the governments and cooperation is to differentiate between diverse sources of eatables at the market level. Further perceived knowledge is constantly observed to be a significant predictor of consumer trust of GM food.^97,98^ It provides ample evidence that prior consumer knowledge helps to build strong intentions^99,100^ regarding GM food. Hence, in both cases, perceived knowledge negatively influences the GM food trust.

Secondly, trust in institutions, trust in technology and revealed information was found to be non-significant predictors of the GM trust in the Chinese context. In the case of revealed information, it is in line with the previous findings of.^101–103^ Whereas, institution trust and trust in technology, the findings are contradictory to the.^104–106^ China ranked number one in institutional trust because Chinese followed the “capitalist system” in this system people rely on institutions for doing everything, in GM food context people are not willing to trust the state institutions as the main source of information in China remains the social media^107^ that contains self-generated opinions and rumors.^48,108^ Further, China is among the top few countries adopting high-tech technologies and related higher trust in technology.^109^ The results show that in food terms, high-tech technologies are not welcomed with a similar passion.^24,110^ The right reason for such maladaptive behavior can be recent food scandals involving cooperative organizations and high-tech technologies that shocked the Chinese society^111^ and lower scientific knowledge.^47^ On the other hand, in the US context, institutional trust and trust in technology were found non-significant against consumer GM food trust. The USA is top of the list in technology introduction and adoption, but consumer behaves differently for high-tech food technologies. The recent social activism in western societies might be a possible reason for such diverse opinion.^112^

The study further incorporates the BRA (perceived benefit-risk analysis) with trust antecedents to enhance the predictive base of the theoretical model. In Chinese data, perceived risk mediates between the revealed information and perceived knowledge because of Chinese social media which is the primary source of GM knowledge in China^110^ and in the virtual world, cynical opinion leaders with nonscientific background lead the anti-GM campaign with the vast following.^113^ On the other hand, the positive and negative attitudes of Chinese consumers are complex and linked with perceived knowledge of science and technology, people’s lifestyles and perceptions about GM food. This is not the only one factor which influences the consumer perception about GM food trust in china there are many such as price, easy availability of GM food in the market, quality, people’s feedback about GM food products. Further, perceived benefits mediate positively between institutional trust, revealed information, perceived knowledge and GM trust to consume except trust in technology in the Chinese context because China is the first country to disclose GM information in terms of labeling in a quest to win consumer trust.^103^ Whereas, revealed information on the GM products provides a clear understanding to the consumers about nutrition values, manufacturing and expiry date and brand positioning that minimize the health concern to reduce the high calories problem without leaving the food preferences. Trust in institutions and on an expert has a strong impact to shape the GM trust to deal with perceived risk and benefits. Trust in institutions using novel technology in food and gene also reduced the risk perception and enhanced the perceived benefits of this gene Technology in food. Whereas often, people used one strategy to manage the lack of knowledge about GM food to seek the opinion of experts they trust^114^ because trust in institutions, perceived knowledge and revealed information reduce the uncertainty and complexity to decide to achieve the GM food trust.

Similarly, in the US case, perceived risk and benefit perception also mediates the relationship between institutional trust, trust in technology, revealed information and GM food trust. USA is one the leading country to producing the multiple GM crops from 1996 to 2017 and also contributing 73.1 million hectares of land and 40% of global GM crops, followed by China 2.8 million hectares,^20,115^ farmer in the USA also rapidly adopting GM crops because of perceived benefits such as productive and financial benefits as compared to China. On the other hand, in the USA, the majority of the consumers are consuming GM food in daily life apart from this perceived risk emerges because food scandals and media controversial talks change the public perception. For instance, on December 4, 2014, an independent nonprofit organization, Intelligence Squared US held a TV discussion on “World is better off with or without GM food” they also included the GM food is safe or has any impact on the environment? At the start of expert debate on GM food, 32% of attendees are in favor of GM food 30% are against, after 100 min debate on this topic attendee’s response change from 32% to 60% in favor and 30 against. This finding is aligning with our outcome to conclude that people’s perception, behavior, attitude and action change in the favor of GM food with time, expert opinion, institutional performance, perceived knowledge and media debates. Whereas, the perceived risk might be reduced to address the public concerns regarding rebuilding the trust in intuitions, trust in technology and promote the beneficial effects of GM food by sufficient revealed information which leads to the GM trust. To the best of our knowledge, this study is first to integrate the trust antecedents and BRA to study GM food trust. These findings highlight the importance of more benefits communication and lesser focus on associated risks.^55,110^

The current study introduces the moderating role of food technology neophobia between the BRA and GM trust. Whereas, neophobia in food technology is explicitly referred to a fear of new or unfamiliar technologies in genetically modified foods that have acquired the intent of customers in both countries to consider the importance of good nutritional values in hygienic foods. Many individuals have specific food preferences that are either appropriate for a healthy body or not; they are consuming in daily life apart from this GM food provide the set revealed information on the GM food product, which assists to the consumer to take proper hygienic, quality food make healthier and smart. The statistical results show that food technology neophobia moderates the relationship between perceived risk and GM food trust in both data set. We found that some consumers highly concerned about GM food because of food scandals, controversies and illegal GM food production some researchers^74,116,117^ also confirmed the consumer concern about “credence qualities” of food technology, such as safety, durability, health, environmental and nature, that can lead to perceived risk, skepticism, and insecurity, especially when consumers lack trust and understanding about novel food technologies.^118^ In the Chinese context, food safety concerns also growing because of some scandals^119^ confirmed that illegal “gutter oil” used in feed additives and cooking which is a common problem with the food chain along with polluted water resulting in oversight of institutions in China. The perceived risk of GM food is getting more negative popularity because of these scandals and practices. Therefore, in China, food technology neophobia negatively contributed to the perceived risk and GM food trust. On the other hand, we found food technology neophobia moderates the relationship between perceived benefits and GM food trust in both data sets. In the Chinese context, food technology neophobia has a moderation effect slightly because the majority of the Chinese population do not have a complete understanding of the GM food even some people did not hear about GM food. Moreover, China is critical country because they contain 20% of the world’s population, 25% of the world’s grain output^119^ with these facts Chinese Government vastly investing the resources in research and development of the technologies to increase the output of the food products and GM food provides the solution to cope up with upcoming hunger problems.^119^ GM food also fulfills the needs, demands and wants consumer perception accordingly. Many consumers are interested in the potential benefits of new food technology because of product quality, appearance, taste, and disease-preventing ability.^120^ In the case of USA, food technology neophobia highly moderates the relationship between perceived benefits GM trust. The USA is one the famous country to producing the GM food and exporting to the other countries; also 90% of soybeans, corn, cotton and canola come from the GM grains in the USA^121^ majority of the USA is perceiving the benefits from the GM food and they also trust on it but some controversies also exit about GM food in the USA. On the other hand, the behavior of food consumption has always been a complicated subject because numerous factors can influence consumer decision making.^68^ Personal traits of consumers are essential characteristics, which have a strong influence to shape the behavior of an individual to take action to accept unfamiliar genetically modified food products.

#### Theoretical Contribution

Present study conclusions have the following theoretical contribution. The statistical results of this study confirm the applicability of trust antecedents, BRA framework as mediators and food technology neophobia as moderating effect in the context of GM trust. PLS-SEM analysis shows that food technology neophobia plays an influential role in framing consumer perception regarding GM trust in both data sets. In both data sets, consumer’s intentions toward GM trust are adversely influenced by the food technology neophobia.

First, this study expands the existing body of literature in consumer food trust and food marketing by providing a new theoretical dimension for predicting GM trust. This will open a new window of opportunity for scholars to investigate the consumer’s behavioral intention in the context of food consumption. Second, the statistical findings of this study validate our argument that the lower level of institutional trust and technology trust will weaken the consumer trust in GM food. Further, the present findings also validate the higher consumer risk perception lower the trust probability and the higher perception of the benefit better the trust.^122^

Last, the current study is comparative nature: to compare the two entirely different cultural, political system, geographical positioning and regulatory agencies. For instance, China has a capitalist system or communist system, which quite different from the democratic system in terms of power-sharing. To compare these two countries on special point, GM food gives new paradigm to the policymakers, governments, public and private companies to make strategies to evaluate the real market situations, financial positioning, import and export, people perception and attitude toward the GM food and agricultural dependencies for predicting the future dominant positioning in the world. These interactions provide different windows of opportunity to businesses, such as to fine-tune their marketing strategies to meet the current aversive behavior of consumers toward GM food.

#### Practical Contribution

Based on the above-mentioned findings, the current investigation has some critical practical implications. First, the world is facing major challenges such as climate change, persistent poverty, overpopulation, hunger challenge of feeding 9.7 billion people by 2050^119^ which will become severe in upcoming years, meanwhile people also demanding the food which will give them good nutrition vales according to own preferences. We address GM food trust, which the vital determinant to manage the aspect mentioned above. Moreover, national and multinational food firms will be better positioned to develop strategies to address consumer needs, improve their product perception and enhance consumer trust by understanding the influential factors based on GM food trust. For instance, consumers who are facing obesity or high cholesterol challenges because of intake of high calories food look toward the better option which provides the variety of preferences and also overcomes these challenges, in this context GM food assists them in making own preferences without leaving the food products with perceived knowledge and reveals information.

Second, national and multinational food firms can improve the level of institutional trust, technology trust, information revealed and knowledge base develop trust and enhance the product reputation. For instance, national and multinational food firms may organize the workshops, seminars, media debate to target the young population which will be the mainstream in upcoming years, as we know to engage the educational and governmental institution in this debate to change the public perception, attitude about GM food and also builds the strong bond between the people and institutions. Business firms can also bring famous personalities compared to scientists to advocate the GM food concept to enhance GM trust. These social personalities can provide an opportunity for individuals to reframe their perception, which in turn, produces GM food trust. This trust also helps the firms to continuously addressing the people concerns about reducing perceived risk and enhances the benefits of GM food.

Third, the finding of the current study also proposes that benefit-risk perception mediates the relation between trust antecedents and GM trust. For example, GM technology developers can design social interaction strategies to providing the opportunity to the individuals to gain better knowledge and communicated more benefits and reduce the concerns, uncertainties and risks. Forth, the prior literature provides evidence that personal experience positively influences consumer trust. Besides the online defense, GM business firms can provide product trails and literature to encourage GM consumption and enhance consumer trust. In addition, by gaining consumer preferences, businesses can redesign GM introductory strategies. Further, the statistical outcome proposes that food technology neophobia adversely moderates the relation between BRA and GM trust. The business managers can increase technology communication to GM consumers to reduce the negative perception of technology involvement in food manufacturing.^56^ To improve GM trust, managers should provide convenient and comfortable communication channels to develop healthy relationships. Literature reveals that food information communication helps to restructure consumer perception.^123^

Finally, the application of high-tech technologies in the food segment is not the only improvement, but it also brings unique psychological experience to consumers. We recommend that businesses consider the potential role of technology when considering the application of food technologies, should be focused on technology communication at the laymen level. Like GM trust, technologies can weaken trust and affect consumers’ relationships.

#### Limitations and Future Directions

Like other research studies, the current study also has a few limitations. These limitations might lead to future research. First, we used cross-sectional data are an appropriate way to test our theoretical model in the dynamic environment because it is collected by structured questionnaires using systematic techniques which are the highly recommended way in social science to gather cognition-based responses from individuals. Researchers may also use these records to compare with unique country sample sizes in the natural environment. Cross-sectional data allow us to add many variables in a dynamic environment to investigate each angle of the conceptual model. We may also consider the longitude data for future research except for cross-sectional data. Longitude data is naturally the same with cross-sectional data but in which data repeatedly collected over a different period. Second, the present study context is restricted to the genetically modified food items consumption trust and moderating role of food technology phobia. This framework can be extended to other controversial products such as robot adoption^124^ and GM medicines.^125^ Further, the data sample is limited to the US and China potential GM consumers. Future researchers can consider more generalized data from multiple data sources with diverse cultures and ethnic intentions. Third, the present study is limited to GM trust antecedents. Future studies may investigate the actual consumption intentions and consider other constructs that may explain the differences between trust and actual consumer intentions.

## Appendix Measurement scale

**Table:**

Institutional Trust		(Verdurme & Viaene, 2003)
1	Food producers have sufficient knowledge and skills to guarantee the safety to food products	
2	Food producers always comply with the regulations related to food safety	
3	Food producers are concerned about the safety and health of consumers	
4	If food producers found to have hidden safety problems in food production, food producers can take the initiative to recall the products	
5	Food producers are honest about the safety of food	
Trust in Technology		^126,127^
6	The GM technology abides by standards and policies (within the industry and universal standards).	
7	In GM technology, legal and technological parameters are adequately addressed to protect me.	
8	I feel confident that GM advances in food science make it safe for me.	
Perceived Knowledge		^128^
9	I’m personally very knowledgeable about GM foods	
10	The average person in China is very knowledgeable about GM foods	
11	The government is very knowledgeable about GM foods	
12	science is very knowledgeable about GM foods	
Revealed Information		^129,130^
13	GM labeling provides correct information on GM foods	
14	GM labeling provides timely information on GM foods	
15	GM labeling provides sufficient information	
16	I am satisfied with the information that GM labeling provides	
Perceived Risk		^131^
17	Applying gene technology in food production will cause environmental hazards.	
18	Genetically modified organisms are likely to interfere with wild species in nature.	
19	Nobody knows the long-term consequences on the environment and human health of applying gene technology in food production.	
20	Applying gene technology in food production will only benefit the producer.	
21	Applying gene technology in food production is unnatural.	
Perceived Benefits		^131^
22	Genetically modified food products will improve the standard of living of future generations.	
23	Genetically modified food products will increase my own and my family’s standard of living.	
24	Genetically modified food products are healthier than other food products.	
25	Genetically modified food products are of better quality foodstuffs than other food products.	
Food Technology Neophobia		^132^
26	New foods are not healthier than traditional foods.	
27	The benefits of new food technologies are often grossly overstated.	
28	There are plenty of tasty foods around, so we do not need to use new food technologies to produce more.	
29	New food technologies decrease the natural quality of food.	
30	New food technologies are unlikely to have long-term adverse health effects.	
31	New food technologies may have long term adverse environmental effects	
32	It can be risky to switch to new food technologies too quickly	
33	Society should not depend heavily on technologies to solve its food problems.	
34	There is no sense of trying out high-tech food products because the ones I eat are already good enough.	
GM Trust		^111,133,134^
35	GM food is trustworthy?	
36	I trust the institutions certifying GM food products	
37	I trust a quality GM food label or logo	
38	GM foods sold in the supermarkets or grocery stores are safe to eat.	
39	GM food meets my expectations	
